# Remarks on Uterine Polypus, with a Case of Extirpation of the Same by the Knife

**Published:** 1843-08-19

**Authors:** John M. Turner

**Affiliations:** Barnwell District, S. C.


### THE MEDICAL EXAMINER,

### And Retrospect of the Medical Sciences.

Vol. VI.] PHILADELPHIA, SATURDAY, AUGUST 19, 1843. [No. 16.

### REMARKS ON UTERINE POLYPUS,

*With a Case of Extirpation of the same by the Knife.*

BY JOHN M. TURNER, M. D.

Different definitions have been given by different writers, in their various works, to designate the term polypus. Thus, Mr. Clarke, in his work on Women, page 243, Vol. 1st, while speaking of uterine polypus, defines it to be "an insensible tumour attached to the internal part of the uterus by a small neck, forming a disease of a very important character." This definition does not exactly correspond with the history given of this complaint by Dr. Denman, who says, page 123, "Some of them hang by small pedicles, and others have a broad basis, especially at their commencement." Nor is it as satisfactory as the definition given by Levret. He makes it "an indolent tumour, more or less salient, resembling a fleshy or fungous excrescence, covered by the membrane from which it takes its rise, and which is of a greater or less thickness."*

One of the most simple definitions of polypus we find given by Dorsey, in his surgical work. He says, "a polypus is a fleshy excrescence, of various density and colour, originating from the lining membrane of a cavity or canal, as the nose, vagina, uterus, rectum, &c.

But we think the definition given by Professor Mütter, in his clinic, in 6th Vol. Medical Examiner, No. 9, the least objectionable. He remarks that, "where a tumour, especially if it be pyriform in its shape, forms in any of the internal cavities which are lined by mucous membrane, it is termed polypus." Its name originated from a very incorrect idea that its radicle, or place of junction, was similar to those of the sepial or marine polypus, by which it attaches itself to the adjacent parts.

Tumours of this nature are occasionally met with in practice, and, we fear, exist more frequently than they are recognised by practitioners, as the case in point will partly go to show. This should lead to a more ample investigation of those cases of uterine affections which we too often consign to their fate, as cancer, corroding ulcer, &c.; for really, the disagreeableness in the nature of a correct investigation of uterine affections, and the small ray of hope that is entertained by the physician himself, together with the feelings of delicacy on the part of patients themselves, generally cause uterine affections to be shamefully neglected, often maltreated, and their relative bearings to other affections, as cause or effect, so little understood, as to leave the practitioner most commonly without the knowledge of sound induction, and consequently, the patient to a loose, imperfect, and unsuccessful treatment, or no treatment at all.

Polypous tumours may grow or sprout out from any part of the uterus, as the fundus, cervix, or os uteri, or even from the vagina; and from this circumstance writers have made their different species. But this appears to us quite an uncalled for distinction, as it is derived entirely from the situation or attachment of the polypous growth, and not from any difference in their organization, nature or texture; therefore it is a mere difference of location. The case which recently fell under our care, a brief description of which we give below, was attached to the cervix uteri; and we are inclined to think, from conversation with several medical friends, and from statistics given by writers on the subject, that the cervix is the part that the pedicle is most often attached to. They have again been classified according to consistency, as the mucous, the fleshy, the fibrous or schirrous, the cartilaginous, and even the osseous. These, too, require almost as little real division of classification as that dependent on location, as the same treatment will pretty nearly suit for all.

These tumours sometimes ulcerate and send forth excrescences; and if they do not thus ulcerate in their own substance, they often produce and keep up such constant irritation in the adjacent parts, as to produce ulceration in some portion of the vagina, and most often in the posterior part, where it lies imbedded in the hollow of the sacrum, eventually producing fatal destruction of the recto-vaginal septum.

The most frequent kind of polypi that are met with, we are inclined to think, is what is denominated the fibrous; they are covered with a production of the inner membrane of the uterus, and, indeed, it seems to proceed or originate from a morbid change or condition of that membrane, and a slow subsequent enlargement of the diseased portion, especially if the woman have borne children. We are under the impression that a hasty delivery of the placenta before uterine efforts come on to produce a natural expulsion, so lacerates the bloodvessels of the lining membrane as to produce irritation, and consequently inflammation, which, owing to the inflammatory condition and sensitiveness of the whole uterine system, may degenerate into a chronic form, and consequently change of structure, that may end in the formation of a polypous tumour. Such, we believe, was the case with our patient.

An extraneous substance, as a polypus, extending from the cavity of the uterus into the vagina, where it comes in contact with the very sensitive and delicate membrane lining the same, produces in it a continual irritation, friction, and incipient inflammation, by which it is made to take on an abnormal action, and pour out continually an increased and morbid secretion, known as leucorrhœa—which is among the first symptoms often of the existence of such a disease. And as the tumour itself gradually increases in size, the bloodvessels of the same, particularly the veins, become more numerous, and at the same time much enlarged, and from their nature, and the constant friction to which they are subjected, become ruptured, from whence exudes sometimes periodically, and at

* Vide Dewees on Women, page 182.

other times observing no regular period, an excessive and debilitating hæmorrhage, which often brings the woman to the point of dissolution. These, together with a sensation of weight, or "bearing down" as patients term it, in the lumbar region, connected often with uterine efforts, which at times produce a partial expulsion of the tumour beyond the os externum, are some of the symptoms that exist in polypous tumours, and which would necessarily lead us to an examination per vaginam; at which time, if it be not very small, and located entirely within the cavity of the uterus, it may be distinctly recognised by the finger to be a smooth tumour, of a pear-like shape, being insensible itself, but producing great tenderness in the lining membrane of the vagina that it lies in contact with. It will also be found adhering firmly to some part of the cavity of the uterus, or os tincæ, or vagina.

Writers, in speaking of its diagnosis, say it may be mistaken for prolapsus uteri or inversio uteri. This, we think, may only happen under two circumstances—either from a want of anatomical knowledge itself, or from a neglect or want of tact in the manual examination. From the first it may be distinguished by the absence of the os tincæ at the lowest part, and want of sensibility in the tumour. In polypus, too, the lowest part is largest; whereas, in prolapsus, it is smallest. In polypus, too, the os uteri is at the upper part, and often surrounds the pedicle like a ring; from the latter it is sometimes more difficult to distinguish. Still, we think the sensitiveness of the tumour in inversio uteri, together with the difficulty in feeling the os uteri, combined with the general symptoms peculiar to that affection, ought to be sufficient to enable us to detect it from such extraneous growths as polypus tumours. But, as our intent in the present instance was more to give a brief sketch of a case that recently fell under our care, with its result, than to write an essay on polypous tumours generally, we will content ourselves with the foregoing remarks. A short notice, however, of the treatment of such affections as laid down by authors, we think, claims a small share of our attention.

Nearly all of the older writers express but one opinion as it regards the nature of the treatment to be adopted in the removal of polypous tumours, and that is, *extirpation by ligature*.

Thus, Dr. John Macintosh, in his work on the Practice of Physic, page 382, 2d Vol., says, "the sooner a ligature is applied the better."

Mr. Burns, in his work on the Principles of Midwifery, and Diseases of Women and Children, 1st Vol., page 107, says, "the only way or method now practised, is to pass a ligature around the base or foot-stalk of polypus, and tighten it so firm as to kill the part below it."

The late Professor Dewees, in his work on Females, while speaking of the different species and treatment, remarks: "all these species have one common remedy, namely, *extirpation by ligature.*"

Of late years, however, both Dupuytren and Velpeau advocate a different plan, namely, that of excision with the knife; the superiority of which I think plain when well executed. The knife I have heard objected to, by some, on account of the danger that a fatal hæmorrhage might ensue. This might, on a superficial view, have sufficient reason to make us dread to undertake the operation. But, then, on a more strict examination of all the parts that compose the pedicle, and that will have to be divided by the knife, we find the largest and majority of bloodvessels to be veins; that it is often from their engorgement or rupture that hæmorrhage takes place during the continuance of the disease; that, too, hæmorrhage from these vessels, when divided, is more easily managed by pressure, aided by styptic applications, than from arteries.

Again: the pedicle to be divided is generally small, and very often of a fibrous nature, through which large arteries do not generally permeate, both of which circumstances would argue against the danger to be apprehended by hæmorrhage. Farther: in the use of the knife the part to which the pedicle is attached, generally the uterus, will have to be drawn down very near or quite to the vulva; when, after the operation, we can immediately apply the strongest styptics, aided with the pressure of the tampon, directly to the incised surface. And to eradicate entirely such doubts, we find, from experience, and the record of what few cases have been published, that where the knife has been used, hæmorrhage has not been the source of fatal termination, but that it has been owing to a consequent inflammation attacking the uterus, and extending from thence to the peritoneum, as was the result of a case that M. Velpeau operated on last March in La Charité; where, he says, the unfortunate sufferer died two days afterwards from peritonitis.*

The knife, then, might be objected to on account of the peritoneal or uterine inflammation that might ensue. Here we would add, that hysteritis and peritonitis often follow the use of the ligature, and are, indeed, we think more apt to occur than when excision is performed by the knife, and for these reasons: that the local sanguineous and purulent discharge from the diseased parts at the time of the operation, and for many days after, acts as a topical depletion, and is much calculated to lessen the irritation then existing, and consequently prevent a propagation or extension of any inflammation that might otherwise ensue. For the purpose of more fully confirming such an idea, and of showing, from an experience in the matter, the advantage of the knife over the ligature, we shall give brief sketch of a case in which we used the knife with complete success.

Case.—Mrs. D——, aged about twenty-one or two years, had borne but one child, and that about sixteen months previous to my seeing her. Her labour was a difficult one, especially in the delivery of the placenta, in which considerable tractive force was used by the midwife. From this accouchment she was a long time recovering, and from it may be dated the commencement of her bad health, which proved to be only the symptoms of a polypous tumour; for from that time leucorrhœal discharges commenced, and a few months after uterine hæmorrhage, to an alarming extent, supervened, sometimes periodically, at other times observing no regular period, until she would often be brought down almost to a complete state of exhaustion, from the great loss of blood; during which time she was under the care of a practitioner who would administer some of the different astringent mixtures, with little or no relief to the great sanguineous discharge.

Other medical advice in the neighbourhood was then sought, but the result was nearly the same; only temporary relief was obtained. At length, her health

* Vide Journal des Connaissances Med. Chir. March, 1843.

continuing to grow worse daily, from the constant vaginal discharges, either leucorrhœal or sanguineous, we were sent for. On our arrival, hearing how much blood had been lost, the tampon naturally suggested itself. (This was in March last.) But, previous to the use of it, on hearing the history of her case related by her husband, I could not satisfactorily account for all the symptoms, and took it to be nothing more than a case of menorrhagia. I consequently resolved, if possible, before prescribing, to investigate the true cause of these discharges by an examination "per vaginam," which being agreed to by our patient, a large polypous tumour was without difficulty detected, growing from the cervix uteri, protruding through the os tincæ, and lying imbedded in the hollow of the sacrum, while the lower part of said tumour rested on the perineum. I immediately had recourse to strong astringent injections (decoction of oak bark and sulph. alumina) into the vagina, followed by the sponge tampon, that was well saturated with a styptic lotion, with cold applications to the pubic region, and strict confinement to the horizontal position.

After some hours the discharge gradually lessened, when I informed the patient and her husband what was the true nature of her complaint; that extirpation was the only resource, without which death was inevitable. I explained at the same time, as well as I could, the nature of the operation, both by ligature and knife, and left her, saying, if she should at any time conclude to undergo an operation, to let me know.

The patient afterwards consulted Dr. P. F. Eve, and Dr. J. A. Eve, the first Professor of Surgery, and the latter Professor of Obstetrics in the Medical College of Georgia, both of whom confirmed my opinion, that its removal was the only means of prolonging her life. They suggested an operation instanter, but to which the patient would not at that time submit.

A few days after the above advice was obtained she placed herself under my care, resolved to undergo any operation I might suggest. At this time the parts were cover d with a continual sanious discharge, that exhaled an unpleasant odor, and the hectic flush could be seen to gradually fill the almost bloodless cheek.

Accordingly, on the 13th day of May, about six weeks from the time I first saw her and explained the nature of her disease, I performed the operation in the following manner. After ordering the rectum and bladder to be evacuated of their contents, a large arm-chair was prepared, the front legs of which were elevated near two inches higher than the posterior, so as to give the patient a half recumbent position, on the front part of which chair she was seated, and an assistant placed at the back part to steady it, and support her head. While thus situated, the thighs being separated from each other, I introduced my right hand, and without much difficulty grasped the tumour; then, by a *gradual*, *slow*, but *steady* traction, succeeded in bringing it out of the os externum. After it was drawn thus far I examined it well, to ascertain as much as possible its nature, which was of the species denominated fibrous; its pedicle was tough, and rather granulated.

After examining well its structure, and attachments to the cervix uteri, I grasped it by its pedicle with the thumb and fingers of my left hand, making them the guide for the tumour, and interposing partly the index finger of the right hand, in which the scalpel was held, between its blade and the uterus—making said finger serve in part as a guide, to prevent the substance of the uterus from being injured; then, by two or three cautious incisions, the pedicle was divided at its very junction with the uterus, which, as soon as done, the elasticity of the ligaments, vagina, and other surrounding connections of the uterus, caused it to slip up towards its natural position. Very little hæmorrhage ensued, however, for fear of which I stuffed into the vagina, and placed immediately in contact with the recent wound, a sponge tampon, that had been previously saturated with a strong solution of acet. plumbi, and sulph. zinci. She was then carefully laid in bed. During the operation she complained much, and approached to near a state of syncope after she was laid down, but from which she was easily aroused by sprinkling with cold water over the face, and stimulants to the nose.

A few hours after the operation our patient complained of much pain in her sides, about the insertion of the broad ligaments of the uterus, owing, as we then supposed, to the great stretch they underwent while the tractive force was used on that organ, and much heat, combined with considerable irritability, in the epigastric region; to allay which, and compose the general nervous irritability, 35 gtt. of the Tincture of Opium was given, which had the happy effect, in the course of a few hours, of putting her in a fine sleep, and allaying the gastric distress.

She remained in that condition until next day, which was the 14th day of May, when the tampon was removed, the bladder emptied, and an astringent injection thrown into the uterus by a female syringe. By this time, too, hæmorrhage had nearly ceased, nor do I think more than four ounces of blood were lost in the whole operation, from its commencement to her discharge.

15th of May. To-day a mild cathartic was administered, which had the effect of producing three or four alvine evacuations. Considerable tenderness also is experienced when pressure is made on the lower part of the abdomen, which produced some dread of peritonitis, but which fortunately never came on to any alarming extent. Diet, too, is ordered to be of the blandest kind; and in the evening, instead of the astringent injection, use freely tepid water, well saturated with Castile soap, thrown in the uterus. In the afternoon of this day some feverish excitement began to appear.

16th May. Much the same as yesterday. Diet same. Vaginal injections same as yesterday evening; also semicupium once per day; discharge from the vagina very much lessened, not more than a teaspoonful in the twenty-four hours. In the evening a mild laxative is given; from this time the abdominal tenderness and febrile symptoms began to subside, and nothing peculiar transpired to prevent her complete recovery.

23d May, it being ten days after the operation, an examination was made with the speculum; the os uteri was found partly closed, but flabby, and easily dilated; the cicatrix had put on a healthy appearance, being healed about one-third around its edges; no discharge from the vagina, only the small portion given out by the wound in going through the process of suppuration. She also is now able to walk about her room, her appetite being good, and digestion perfect.

On the fifteenth day after the operation, an examination being made per vaginam, the sore was still healing, and very little tenderness; the womb, however, remains rather low down in the vagina, which is replaced in its natural position, and confinement to bed in a horizontal posture for a few days was complied with, and cold astringent injections resorted to for the purpose of strengthening the debilitated vagina. This being complied with, and no unfavourable symptom presenting itself, she was discharged, about twenty days after the operation, with no hæmorrhage or leucorrhœal discharge from the vagina; a small portion of pus, however, might be seen occasionally of a morning from the vagina as the sore healed. Her constitution being much enfeebled from the frequent loss of blood sustained at intervals for the year past, I gave her, when dismissing her, a box of pills, composed of the Precip. Carb. Ferri, two of which was to be taken daily; and recommended mild, but nourishing diet.

About six weeks from the operation she menstruated in small quantity; and at her next regular period it again appeared in larger quantity, putting on more of a healthy appearance, from which I infer that the uterus is gradually gaining its natural healthy tone.

Her general health has improved as much as could be looked for in the small space of two and a half months, though the pallid cheek has not yet entirely regained its former blush of "perfect health." Still, we feel no doubt, as the source of irritation is removed, and morbid action eradicated from the uterine system generally, and a tonic reaction already commenced, and progressed to this term, (two and a half months,) that it only requires a few more months to completely confirm her general health.

The polypus was about the size of a large turkey egg, and of a pyriform shape; it was a fibrous substance, of a smooth, pearly lustre, covered with a thin layer of membrane, similar to that which lines the uterine cavity. From the resistance the pedicle gave the edge of the scalpel in the operation, and its toughness generally in making incisions afterward, I apprehend it would have taken a ligature many days to have completed what the knife did in a few seconds. When this is considered, together with the tediousness of the ligature, and prolonged suffering, and more than double inconvenience to the practitioner, and equal, if not more safety to the patient, ought we not, then, in generality of cases, give the knife the preference?

*Barnwell District, S. C.*, August 2, 1843.

